# Case Report: Advantages of High-Resolution MRI in Evaluating the Efficacy of Drug Therapy for Intracranial Atherosclerotic Plaques

**DOI:** 10.3389/fnagi.2022.804074

**Published:** 2022-04-27

**Authors:** Tao Zheng, Lei Liu, Li Li, Yang Gao, Runcai Guo, Zhi Zhou, Zunjing Liu, Kunpeng Liu

**Affiliations:** ^1^Graduate School, Beijing University of Chinese Medicine, Beijing, China; ^2^Department of Neurology, China-Japan Friendship Hospital, Beijing, China; ^3^Department of Anesthesiology, Beijing Friendship Hospital, Capital Medical University, Beijing, China; ^4^Department of Radiology, China-Japan Friendship Hospital, Beijing, China; ^5^Department of Anesthesiology, Peking University International Hospital, Beijing, China

**Keywords:** high-resolution MRI, advantages, evaluating, intracranial atherosclerotic plaques, reversal

## Abstract

Intracranial atherosclerotic stenosis is one of the main causes of ischemic stroke and transient ischemic attack. High-resolution magnetic resonance imaging allows us to directly observe the intracranial artery wall, accurately assess the condition of the vascular wall, and quantitatively analyze the vascular wall and intracranial atherosclerotic plaque load. We report a case of acute cerebral infarction with left middle cerebral artery stenosis. During the first 3 weeks, the patient was treated with aspirin 100 mg and clopidogrel 75 mg daily. Afterwards, the patient continued to be given aspirin, and cilostazol 100 mg twice daily was given instead of clopidogrel. After 24 months of follow-up, we observed a significant reversal of intracranial atherosclerotic plaque using high-resolution MRI (HR-MRI) and discussed the advantages of HR-MRI in evaluating drug therapy for intracranial atherosclerotic plaque.

## Introduction

Intracranial atherosclerotic stenosis (ICAS) is one of the main causes of ischemic stroke and transient ischemic attack (TIA). Stroke is also a major cause of death and disability worldwide (Rahman et al., 2021). Active intervention in plaque progression and prevention of stroke are the main objectives of current drug therapy. Most existing studies evaluate the efficacy of drugs in the treatment of intracranial atherosclerotic plaques based only on changes in lumen, while few studies evaluate the plaques' diameter of the stenosis, vascular area, lumen area, wall area, stenosis rate, remodeling index, wall area index, and normalized wall index using HR-MRI (Chen et al., 2018; Chung et al., 2020; Shi et al., 2021). We report a case of acute cerebral infarction with left middle cerebral artery stenosis. During the first 3 weeks, the patient was treated with aspirin 100 mg and clopidogrel 75 mg daily. Afterwards, the patient continued to be given aspirin, and cilostazol 100 mg twice daily was given instead of clopidogrel. After 24 months of follow-up, we observed a significant reversal of intracranial atherosclerotic plaque using high-resolution MRI (HR-MRI) and discussed the advantages of HR-MRI in evaluating drug therapy for intracranial atherosclerotic plaque.

## Case Description

**Figure 1:**
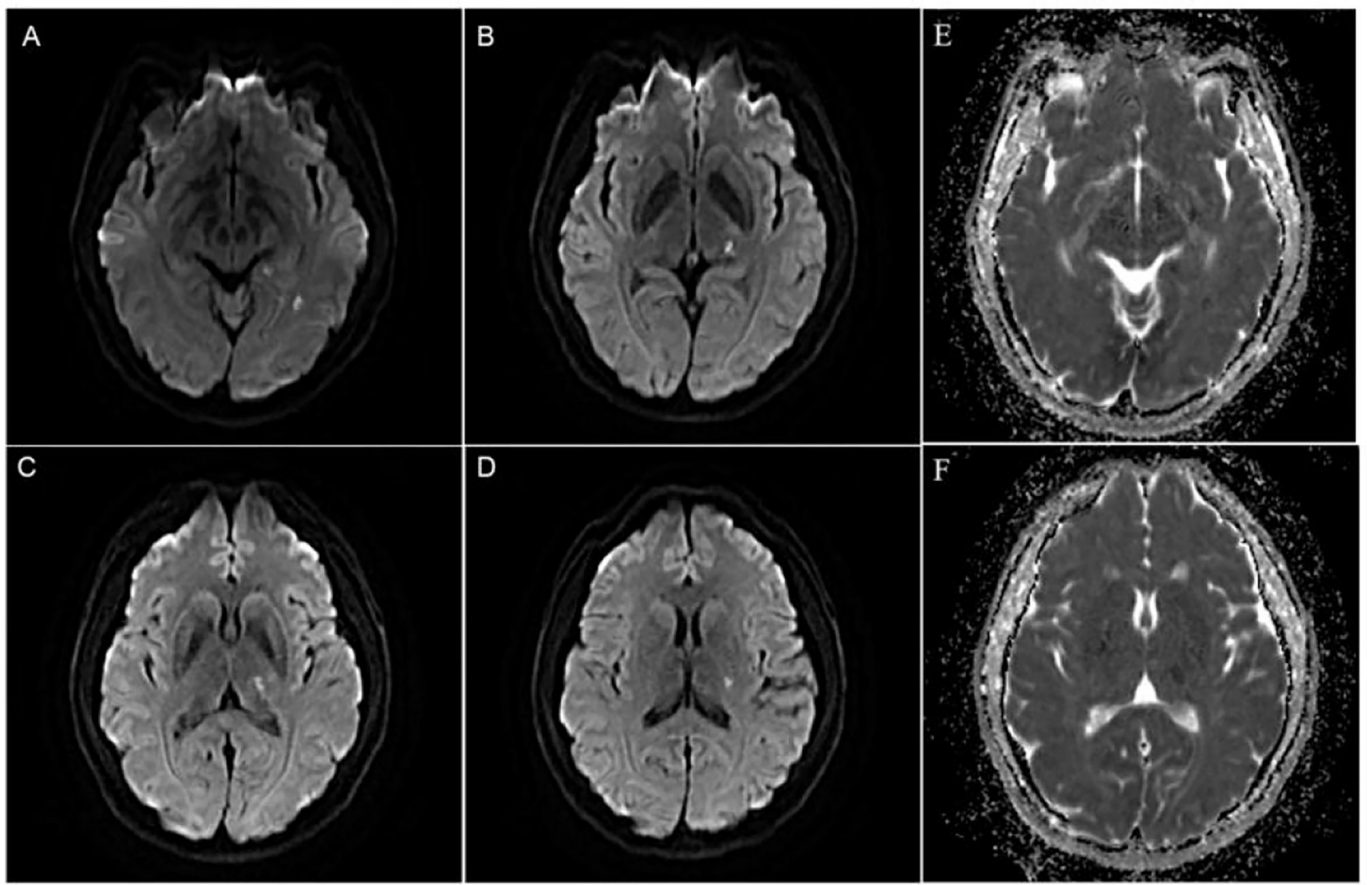
**(A–D)** Diffusion weighted imaging(DWI) showed multiple and small areas of acute infarction on the left basal ganglia, thalamus, and temporal lobe. **(E,F)** ADC image showed multiple and small areas of low signal areas on the left basal ganglia, thalamus, and temporal lobe.

**Figure 2:**
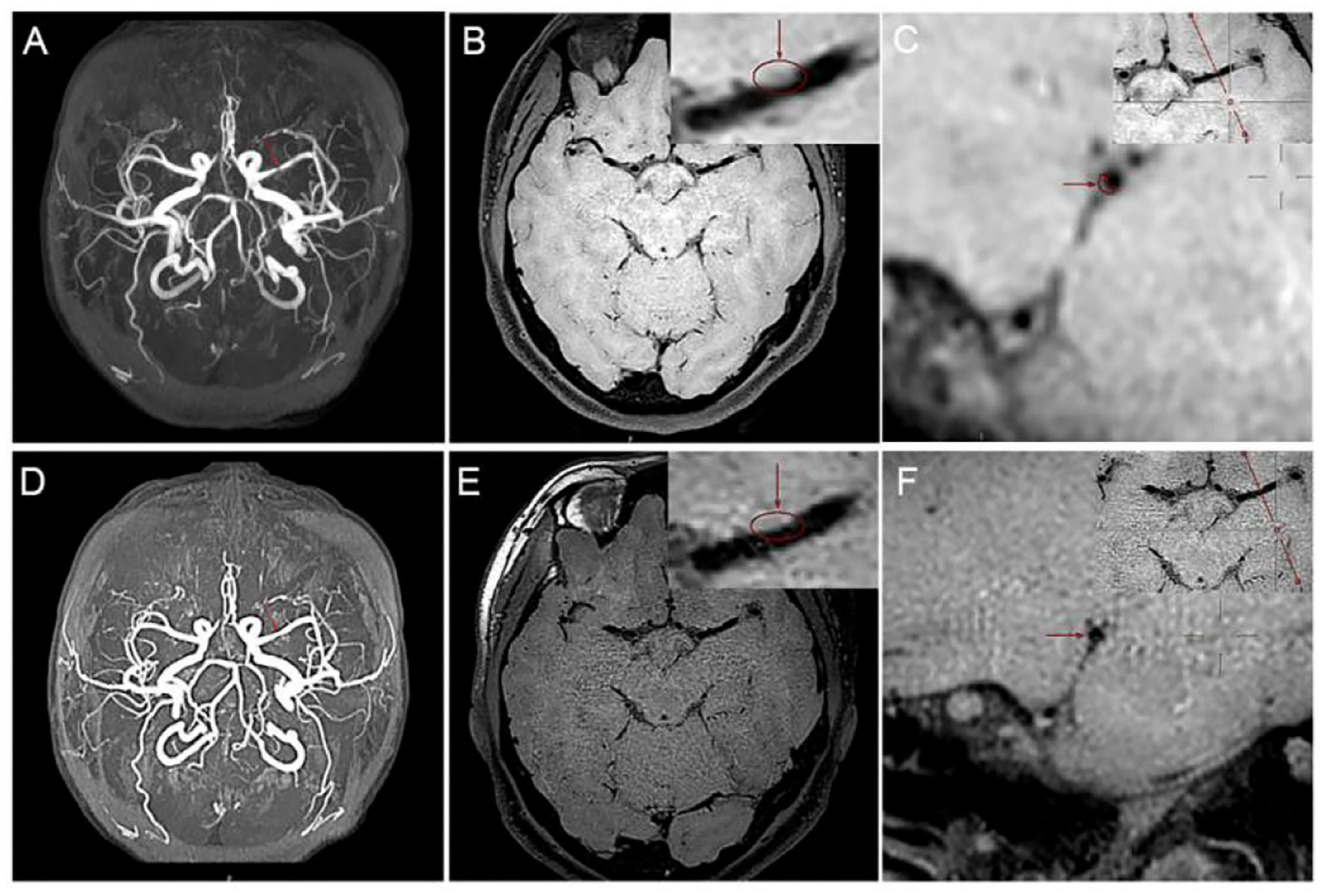
High-resolution MRI (HR-MRI) examination at hospitalization. **(A)** Time of flight (TOF) magnetic resonance angiography (MRA) showed severe M1 stenosis in the left middle cerebral artery (MCA). **(B)** T1-weighted volume isotropic turbo spin-echo acquisition (T1W-VISTA) showed the location of the plaque and thickens the eccentric canal wall. T1W-VISTA also showed no significant enhancement on the plaque or surface, similar to the surrounding tissue signals, indicating no thrombosis on the surface of the plaque. **(C)** 3D-VISTA reconstruction was carried out at the maximal-lumen-narrowing site, and the rough shape of the patch was hand-drawn. HR-MRI examination after 24 months. **(D)** TOF MRA showed that the degree of lumen stenosis was significantly reduced. **(E)** T1W-VISTA showed that there were still some plaques on the tube wall, and the lumen was slightly narrow. **(F)** 3D-VISTA reconstruction showed the diameter of the tube was significantly larger.

A 46-year-old Chinese female presented with a sudden-onset right-sided weakness and numbness 1 day ago. She denied diplopia, visual field change, disturbance of consciousness, headache, and dizziness. She also denied any history of hypertension, hyperlipidemia, and others. There are no obvious abnormalities in the patient's personal and family history. In the emergency room, the patient's blood pressure was 163/85 mmHg. On neurological examination, the muscle strength was IV, the sensation of pinprick was impaired, and Babinski sign was positive in the right limb. The routine laboratory examinations after admission showed that low density lipoprotein cholesterol was 3.47 mmol/L, triglycerides was 1.79 mmol/L, and the rest of the routine laboratory examinations showed no obvious abnormalities. Head MRI showed multiple small areas of acute infarction on the left basal ganglia, thalamus, and temporal lobe (Figures 1A–F). During the first 3 weeks, the patient was treated with aspirin 100 mg daily and clopidogrel 75 mg daily for antiplatelet therapy. Afterwards, the patient continued to be given aspirin, and cilostazol 100 mg twice daily was given instead of clopidogrel. The reason for using cilostazol was that we referred to the CSPS.com trial (see Section Discussion) (Toyoda et al., 2019). During the follow-up, rosuvastatin 10 mg daily and amlodipine 10 mg daily was continued. During the follow-up, the lipid control objectives were as follows: LDL-C ≤ 1.8 mmol/L, systolic blood pressure ≤ 140 mmHg, without complications or neurological deficits. HR-MRI examinations at hospitalization showed moderate to severe stenosis of the M1 section of the left middle cerebral artery (MCA; Figures 2A–C). The reexamination result of HR-MRI at 24 months was better, during which the lumen was slightly narrowed and the wall was slightly thickened in the same place (Figures 2D–F).

## Methods

**Figure 3:**
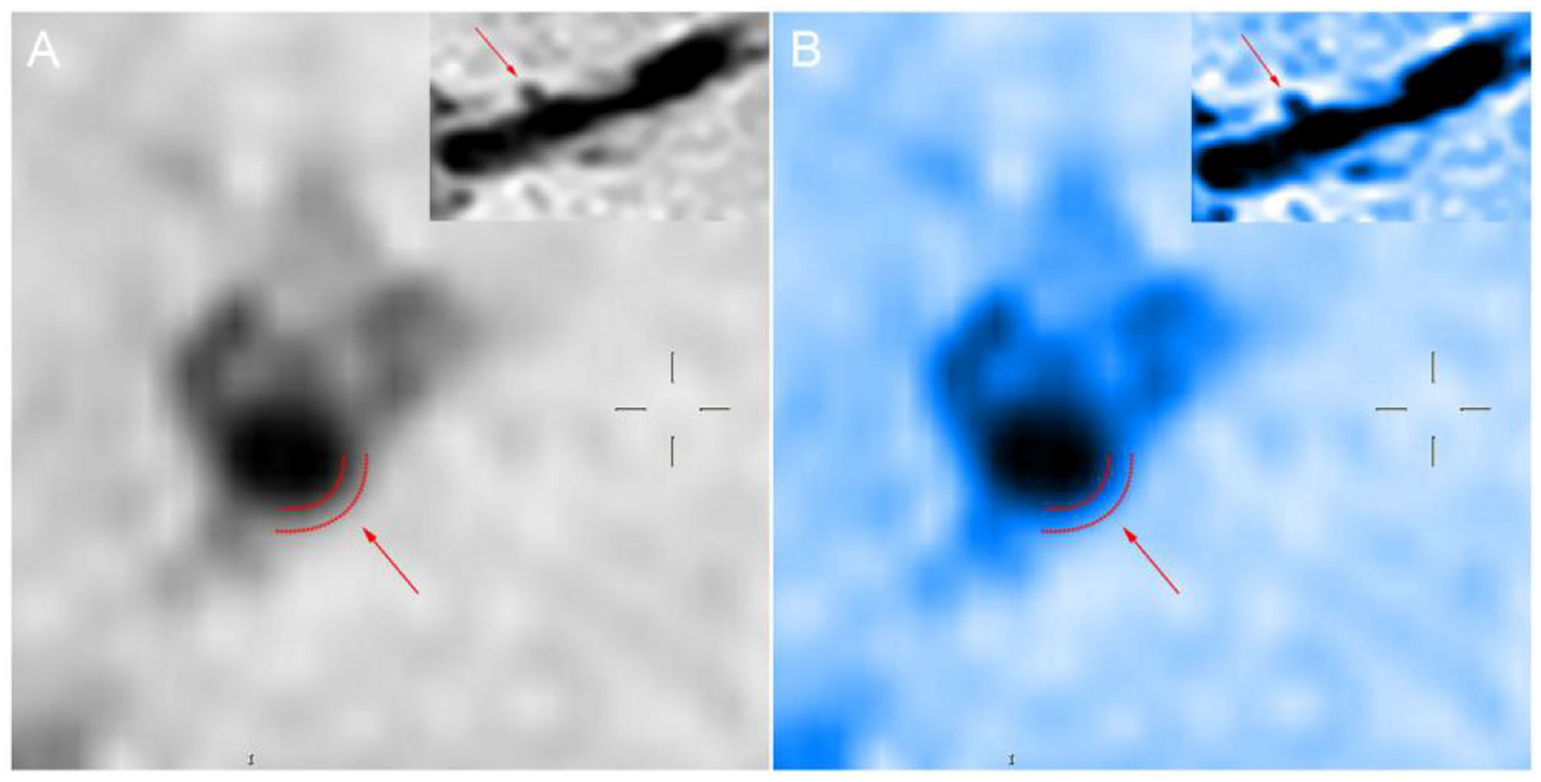
Panels **(A,B)** show the sagittal position of the perforating artery as shown by the arrow at the upper right. The boundary delineated by the dashed line indicated by the arrow in the larger image is the rough outline of the vessel wall.

The high-resolution MRI used in our study was a 3.0 T MRI scanner (Ingenia; Philips Healthcare, Best, The Netherlands) with a 15-channel phased-array coil. Our imaging results were diagnosed by two radiologists with more than 5 years of experience in HR-MRI diagnosis. Three-dimensional (3D) volume isotropic turbo spin-echo acquisition (VISTA) images were obtained by axial plane scanning of the major intracranial arteries with the following parameters: repetition time/echo time, 1,300/36 ms; field of view, 140 × 200 × 105 mm^3^; matrix, 280 × 332 × 210; number of excitations, 2. Acquisition voxel volume was 0.5 × 0.6 × 0.5 mm^3^, and reconstruction voxel volume was 0.5 × 0.5 × 0.5 mm^3^. Axial plane images were automatically constructed with a slice thickness of 0.5 mm. The 3D-VISTA scan time was approximately 5 min. We selected time of flight (TOF) magnetic resonance angiography (MRA) images and 3D-VISTA images. MRA scan was mainly used to determine the location and degree of MCA stenosis and to angle the reconstruction plane of 3D-VISTA images to ensure that all cross-sections were perpendicular to the long axis of the MCA. The cross section of the MCA was divided into four quadrants: upper, lower, ventral, and dorsal quadrant. Moreover, changing the color map of the image from the traditional gray to sky blue will make the outline and thickness of the blood vessel wall appear more clearly. As shown in Figure 3, after we changed the color mapping of digital picture archiving and communication (PACS) workstation from gray scale to sky blue, the contour of blood vessel wall in Figure 3B was clearer than in Figure 3A.

We semi-automatically obtained the vessel diameter, wall thickness, vessel area (VA), and lumen area (LA) of the maximal-lumen-narrowing (MLN) site and the reference site by using the measurement tool on PACS. The calculation formula of the wall area (WA) was WA=VA-LA. The vessels in the non-stenosis segment of the proximal MCA were used as reference points, and the reference wall area and lumen area were measured. The degree of stenosis was computed as follows: degree of stenosis = (1 – LA _MLN_ / LA _reference_) ×100%. The remodeling index (RI) was defined as VA _MLN_ / VA _reference_. The wall area index (WAI) was defined as WA _MLN_ / WA _reference_. The normalized wall index (NWI) was defined as WA _MLN_ / (WA _MLN_+LA _MLN_) ×100%. RI ≤ 0.95 was defined as negative remodeling (NR). Lastly, 0.95 < RI <1.05 was defined as intermediate remodeling (IR), and RI ≥ 1.05 was defined as positive remodeling (PR).

## Results

After 24 months of follow-up, we found that the diameter of the stenosis increased from 1.08 to 1.90 mm, the LA increased from 1.13 to 2.56 mm^2^, the WA decreased from 4.35 to 2.92 mm^2^, the stenosis rate on HR-MRI decreased from 49.53 to 11.21%, the RI increased from 39.65 to 89.82%, the WAI decreased from 1.14 to 0.76, and the NWI decreased from 79.38 to 53.28%, as shown in Table 1.

**Table 1:** Changes of various indexes before and after follow-up.

	**D (mm)**	**VA (mm^**2**^)**	**LA (mm^**2**^)**	**WA (mm^**2**^)**	**R (%)**	**RI (%)**	**WAI**	**NWI (%)**
HR-MRI at hospitalization	1.08	5.48	1.13	4.35	49.53	39.65	1.14	79.38
HR-MRI after 24 months	1.90	5.48	2.56	2.92	11.21	89.82	0.76	53.28

*HR-MRI, High-resolution MRI; D, The diameter of the maximal-lumen-narrowing site; VA, Vascular area; LA, Lumen area; WA, Wall area; R, Stenosis rate; RI, Remodeling index; WAI, Wall area index; NWI, Normalized wall index*.

## Discussion

How to more accurately and more safely evaluate the efficacy of drug therapy for intracranial atherosclerotic plaque is one of the problems that need to be solved in clinical practice. CT angiography (CTA), MRA, and digital subtraction angiography (DSA) help us assess intracranial atherosclerotic stenosis by primarily providing residual lumen diameter. The methods mentioned above do not provide us with information about vascular walls, atherosclerotic plaques, etc. HR-MRI can directly observe the vascular wall and lumen and perform quantitative and qualitative analysis of plaque, thus providing more information of clinical value. MRA may exaggerate the extent of lumen narrowing. For example, the arrow in Figure 2A shows severe stenosis in the M1 segment of the left MCA on MRA, whereas Figure 2B shows approximately moderate stenosis on HR-MRI. Through quantitative analysis, we calculated that the stenosis of the left MCA was approximately 49.53%, which was approximately moderate and not far from the stenosis we estimated on T1W-VISTA compared to the severe stenosis observed on MRA. This also confirms that MRA tend to overestimate the stenosis of blood vessels. Previous studies have shown that CTA may also exaggerate lumen stenosis (Liu et al., 2013).

**Figure 4:**
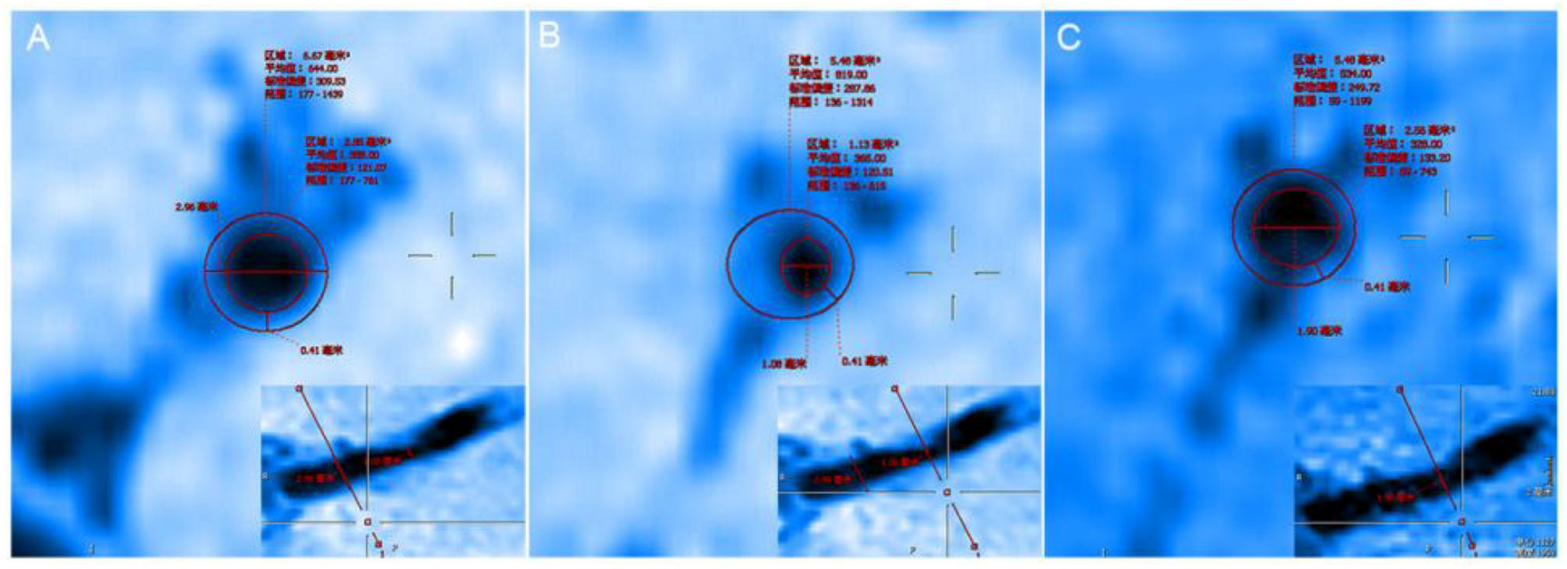
**(A)** The proximal vessels serve as the reference site. **(B)** The maximal-lumen-narrowing site before follow-up. **(C)** The maximal-lumen-narrowing site after follow-up.

In evaluating the effects of drug therapy on intracranial atherosclerotic plaque, the changes of plaque and vascular wall should be paid attention to in addition to lumen. However, MRA, CTA, and DSA cannot provide us with information about plaques and vascular walls. In addition to stenosis, HR-MRI can also measure wall thickness, plaque volume, LA, WA, RI, WAI, NWI, etc. WAI and NWI are commonly used indexes to analyze plaque load, and their calculation methods are shown in the method section of this paper. Large culprit plaque load was independently associated with recurrent acute stroke (Sun et al., 2021). Plaque load can also be used to assess plaque size and drug efficacy. We calculated the WAI and NWI to compare plaque load before and after follow-up, as shown in Figure 4. The WAI decreased from the initial value of 1.14 to 0.74, and the NWI was approximately 79.38% before and 53.28% after the follow-up, significantly decreased by 26.1%. All the above findings indicate that the plaque load was significantly reduced under the intervention of drugs. Although both methods can describe plaque load, the latter is increasingly accepted by the majority of scholars and is regarded as one of the best indicators for evaluating plaque load at present. The main reason is that WAI is calculated according to the ratio of the WA of the MLN site to the WA of the proximal non-stenosis site, which is relatively subjective. However, NWI is based on the ratio of the wall area of the MLN site, which is relatively objective and has certain reference value. The RI is calculated by the wall area, which can be used to judge the stability of plaque. It is mainly divided into positive remodeling and negative remodeling. Positive remodeling is often associated with plaque instability and is prone to cause acute ischemic stroke (Zhang et al., 2017). For stroke patients with positive remodeling, luminal imaging at this time often shows normal or mild stenosis, and the pathological mechanism of stroke is difficult to explain. HR-MRI can help us study the mechanism of infarction in such patients with positive remodeling (Sun et al., 2018).

The duration of treatment and the combination of drugs may be correlated with whether the vascular remodeling index changes significantly. In Chung and Shi's study, significant changes in the vascular RI were not found (Chung et al., 2020; Shi et al., 2021). Particularly, Chung found no significant change in the RI after intensive statin treatment (1.09 ± 0.35 vs. 1.03 ± 0.30; *p* = 0.195) (Chung et al., 2020). In our study, the vascular RI before follow-up was 0.3965, which was much less than 0.95. After follow-up, the new RI was 0.8982, which was very close to the range of normal vascular RI. Prolonged statin therapy may make the plaque more stable and may also bring the remodeling index back into the normal range. The reason may be that the duration of intensive statin therapy (6 months) observed in Chung's study was relatively short. Long-term and appropriate antiplatelet therapy is an indispensable link to reduce clinical events. This was a patient with symptomatic intracranial artery stenosis who is at high risk of recurrent ischemic stroke. However, the patient's DWI showed a small ischemic focus, and the patient was at a low risk of bleeding after taking antiplatelet drugs. The reason we chose cilostazol and aspirin long-term combination therapy is that we referred to the “CSPS.com” trial in Japan, which showed that for high-risk stroke patients, long-term combination therapy of cilostazol with aspirin or clopidogrel had a lower risk of ischemic stroke recurrence (Toyoda et al., 2019). There was no significant increase in the risk of serious or life-threatening bleeding. Other studies confirm this (Lin et al., 2021). Previous studies have found that cilostazol provides benefits after 60–90 days of treatment (de Havenon et al., 2021). In addition to inhibiting platelet aggregation, cilotazol can also promote blood vessel remodeling and lowering of blood lipid levels, among others. Some studies found that cilostazol can stabilize or reverse plaque effects. For example, Lee et al. found that after long-term treatment with cilostazol, carotid plaque volume and intima thickness were significantly reduced, leading to vascular remodeling (Lee et al., 2020). In our study, the reversal of plaque in patients may be related to the use of cilostazol.

## Conclusion

High-resolution MRI is one of the more intuitive and non-invasive auxiliary examinations. Through quantitative analysis of vascular wall and plaque, HR-MRI can help us compare the effects of drugs on delaying or reversing intracranial atherosclerotic plaque from multiple perspectives.

## Data Availability Statement

The original contributions presented in the study are included in the article/supplementary material, further inquiries can be directed to the corresponding author/s.

## Ethics Statement

The studies involving human participants were reviewed and approved by the Ethics Committee of clinical trials of drugs or devices in China-Japan Friendship Hospital. The patients/participants provided their written informed consent to participate in this study.

## Author Contributions

TZ contributed to paper conception, data collection, analysis and interpretation, literature review, and paper drafting. LLiu, LLi, YG, RG, and ZZ contributed to data collection, analysis and interpretation, literature review, and paper drafting. ZL and KL contributed to paper conception, the supervision of data analysis and interpretation, and critically reviewed the paper. All authors contributed to the article and approved the submitted version.

## Funding

This study was supported by the National Natural Science Foundation of China (No. 52073310) and Elite Medical Professionals project of China-Japan Friendship Hospital (NO. ZRJY2021-BJ03).

## Conflict of Interest

The authors declare that the research was conducted in the absence of any commercial or financial relationships that could be construed as a potential conflict of interest.

## Publisher's Note

All claims expressed in this article are solely those of the authors and do not necessarily represent those of their affiliated organizations, or those of the publisher, the editors and the reviewers. Any product that may be evaluated in this article, or claim that may be made by its manufacturer, is not guaranteed or endorsed by the publisher.
